# From Single Strains to Synthetic Bacterial Communities: Microbial Remediation in Saline–A-Alkali Soil

**DOI:** 10.3390/life16060938

**Published:** 2026-06-02

**Authors:** Juanjuan Wang, Wen Huang, Jiaying Cai, Hengjia Zhang, Xiaoqing Qian

**Affiliations:** 1 Key Laboratory of Arable Land Quality Monitoring and Evaluation, Yangzhou University, Ministry of Agriculture and Rural Affairs, Yangzhou 225127, China; wangjuanjuan@yzu.edu.cn (J.W.); wwen991019@163.com (W.H.); dou1131404560@163.com (J.C.); nibbleszed@outlook.com (H.Z.); 2 Jiangsu Collaborative Innovation Centre for Solid Organic Waste Resource Utilization, Nanjing 210095, China

**Keywords:** saline–alkali soil, microbial agents, plant growth-promoting bacteria, soil health, sustainable agriculture

## Abstract

Global salinization affects approximately one billion hectares of land in more than 100 countries, posing a severe threat to food security and ecosystem sustainability. Microbial remediation using plant growth-promoting microorganisms offers an eco-friendly alternative to physicochemical methods. However, bridging the gap between laboratory cultivation of single strains and field-scale application of synthetic microbial communities (SynComs) remains difficult, owing to inconsistent efficacy and a lack of unified design frameworks. This review examines the evolution from single strains to rationally designed SynComs for saline soil remediation. A ‘structure–function–mechanism’ framework is proposed, integrating five core microbial modules, namely ion regulation and osmotic stabilization, ethylene and phytohormone modulation, antioxidant activation, nutrient cycle activation, and systemic resistance induction. The review elucidates key determinants of synthetic community success, including functional complementarity, strain compatibility, and host–environment matching, while revealing a marked quantitative gap between controlled experiments and field performance. Key bottlenecks are identified, including the lack of high-throughput compatibility screening, poorly quantified long-term ecological risks, and the absence of standardized application guidelines across agro-ecological zones. Finally, emerging avenues are discussed, such as microbial–microalgal symbiosis and AI-assisted design, outlining a roadmap for next-generation smart microbial products integrated into climate-resilient farming systems.

## 1. Introduction

Global salinization affects approximately one billion hectares of land in more than 100 countries. Salt-affected soils lead to yield reductions of 50–70% in sensitive species and degrade over 20% of the world’s irrigated agricultural lands (FAO and ITPS, 2015). Under ongoing climate change scenarios, characterized by sea-level rise and increased evaporation, the area subjected to secondary salinization is projected to expand considerably. This will directly compromise food security and ecological stability [1].

Saline–alkali stress hinders plant growth at multiple levels, including osmotic stress, ionic toxicity, nutrient imbalance, and oxidative damage, while also driving structural-functional degradation in soil microbial communities [2,3]. Soil ecosystem functioning is governed by the interplay between abiotic properties (e.g., pH, texture, redox potential, and water potential) and biotic components. Abiotic factors define the ecological niche, whereas microorganisms act as core mediators of key processes, such as nutrient cycling, organic matter transformation, soil aggregation, and stress response regulation, with their function being strictly shaped by the surrounding physicochemical environment [3,4].

Harnessing beneficial microorganisms (i.e., microbial inoculants) for soil improvement represents a highly feasible, nature-based solution [3,5]. Recent research has shifted from screening for single, highly effective plant-promoting strains to synthetic microbial communities (SynComs). SynComs are distinguished from natural assemblages by their predictable functional traits and stable interspecific relationships [6,7,8]. Contemporary findings suggest that restoration outcomes depend not merely on the metabolic capacity of individual strains, but primarily on the complex network of interactions among microbes, as well as their interplay with host plants, the resident microbiome, and the broader edaphic environment [6,9].

The superior performance of synthetic communities over single strains is rooted in three core ecological assembly mechanisms: first, niche complementarity, whereby strains occupy different rhizosphere niches and perform specialized functions (e.g., nitrogen fixation, phosphorus solubilization) to avoid competition [6,9]; second, metabolic cross-feeding, in which strains exchange intermediate metabolites (e.g., amino acids, vitamins) to reduce metabolic costs; and third, quorum sensing, which prevents antagonistic responses triggered by inter-strain chemical signaling [8]. These mechanisms explain why synthetic communities exhibit higher colonization stability and remediation efficiency in complex field environments.

Previous reviews have summarized the functional characteristics of salt-tolerant plant growth-promoting bacteria [4], but most focus on single-strain mechanisms and do not address the paradigm shift toward SynComs. The general SynCom design principles for agriculture were discussed [6,7] but the specific constraints of salinity-alkalinity stress, or framework linking community structure, functional traits, and plant stress-response pathways were not overlooked. Furthermore, few reviews have systematically examined the technical bottlenecks that hinder lab-to-field transition, particularly the lack of comparative efficacy data across different saline–alkali soil types and economic feasibility assessments.

Although beneficial effects of microbial inoculants are frequently demonstrated under controlled laboratory conditions, field-level efficacy often remains inconsistent. Existing studies lack a unifying framework to consolidate disparate microbial functional knowledge, elucidate strain–environment interactions, and guide the rational assembly of remediation consortia [6,8,9]. This review traces the evolution of microbial remediation strategies for saline–alkali soils, from single-strain inoculants to rationally designed SynComs, synthesizes current understanding of their functional mechanisms, research gaps, and future directions, and provides a systematic roadmap for translating SynCom research into large-scale application.

This review uses the Web of science as database, with the search period of 2020–2026 and the keyword combination of “saline–alkali”, “soil” “microbial agent”, aims to: (1) clarify the evolutionary logic, advantages and limitations of different microbial remediation strategies from single-strain agents to SynComs, based on quantitative comparative data from published studies; (2) construct a unified “structure-function-mechanism” framework to organize reported microbial resources and their action pathways for saline–alkali stress alleviation; and (3) identify the key technical bottlenecks restricting the lab-to-field translation of these technologies, and propose targeted future research directions to promote the practical application of microbial remediation technologies.

Building on this paradigm shift, the following section examines the evolution of microbial agent strategies, comparing the strengths and limitations of different technical approaches to lay the foundation for subsequent mechanistic analysis.

## 2. Strategies for the Application of Microbial Agents

The evolution of microbial remediation strategies for saline–alkali soil has transitioned from simple inoculation to complex, ecology-driven designs. As summarized in Table 1, current approaches can be categorized into three progressive stages: the use of Single-function strains, the assembly of Synthetic microbial communities (SynComs), and the integrated SynComs + Soil Amendment synergy.

Early studies focused primarily on identifying and verifying beneficial individual strains [6,7,8]. However, the application efficiency in fields were not consistent, as there might exist competition from native microbes [10,11,12,13,14,15,16]. To address these technical constraints, rational design of SynCom has emerged as a major research focus. Two primary strategies are employed: functionally complementary strains are assembled to form a versatile remediation system [17], or compatible, functionally differentiated members are screened to construct compact, stable, and efficient communities [6,9].

In addition to the configuration of complex strains, another strategy to enhance the repair effect by combining microbial agents with organic or inorganic amendements [2,17,18,19,20,21,22,23]. In order to create a suitable microenvironment for the microorganisms, organic soil conditioners like biochar, humic acid, and straw play a key supporting role. They supply added microbes with the necessary habitats, food sources, and nutrients, while also buffering them from environmental stress. This combination of microbial agents and organic conditioners has been shown to work better than using either one alone. The joint approach is more effective at lowering soil salt and alkali, raising nutrient levels, boosting soil enzyme activity, and helping to form stable soil aggregates [2,17,18,19,20,21,22,23]. Moreover, the soil’s microbial community was shifted toward groups with better functions [21]. Studies show that nanomaterials, like silicon nanoparticles, can reduce sodium ion uptake via physical barriers and act as carriers for plant-promoting bacteria colonization, synergistically enhancing plant stress resistance [22].

**Figure 1:**
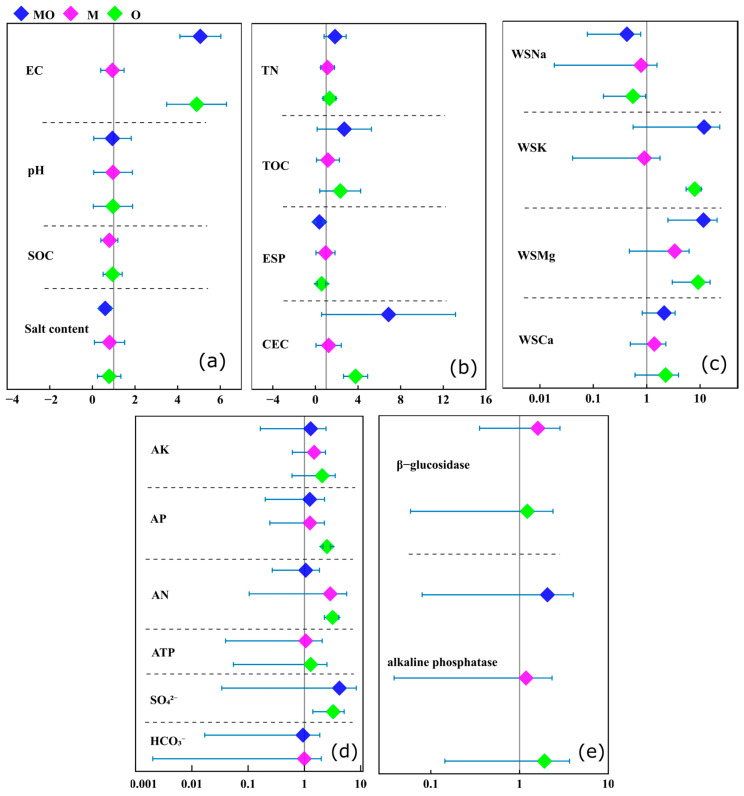
Effects of organic fertilizer (O), microbial fertilizer (M), and combined application of organic fertilizer and microbial fertilizer (MO) on the physicochemical properties of saline soil (**a**–**d**): soil physicochemical properties; (**e**): soil enzyme activities. SOC: soil organic carbon; TN: total nitrogen; TOC: total organic carbon; ESP: exchangeable sodium percentage; CEC: cation exchange capacity; WSNa: water soluble sodium; WSCa: water soluble calcium; WSMg: water soluble magnesium; WSK: water soluble potassium; AK: available potassium; AP: available phosphorus; AN: available nitrogen. Note: Figure 1 and Figure 2 were achieved by integrating data from 61 recently published papers. The calculation steps and the literature are listed in Supplementary Materials (S1).

Comparative analysis of amendment effects on saline soil properties is further conducted. Application of organic fertilizer, microbial inoculant, and their combination significantly altered soil electrical conductivity, with the combined treatment exhibiting the optimal amelioration effect. No significant effect on soil pH, organic carbon and salt content was observed. (Figure 1a). The combined treatment produced the maximum increase in cation exchange capacity (CEC). Organic fertilizer alone also markedly increased CEC, while microbial agent alone had no significant effect. Soil carbon and nitrogen pools and exchangeable sodium percentage (ESP) were slightly affected (Figure 1b). Organic fertilizer, regardless of microbial addition, effectively increased soil water-soluble K, Mg and Ca, whereas microbial inoculant had no influence on these parameters. All treatments failed to significantly change water-soluble sodium (WSNa) (Figure 1c). Organic fertilizer alone significantly increased available P and available N, while soil available K remained unchanged across all treatments. Organic fertilizer application and the combined treatment both elevated SO_4_^2−^ content. Soil ATP activity and bicarbonate (HCO_3_^−^) content were not affected (Figure 1d). Soil alkaline phosphatase activity was increased by the combination treatment, and β-glucosidase activity increased by microbial agent to some degree (Figure 1e).

**Figure 2:**
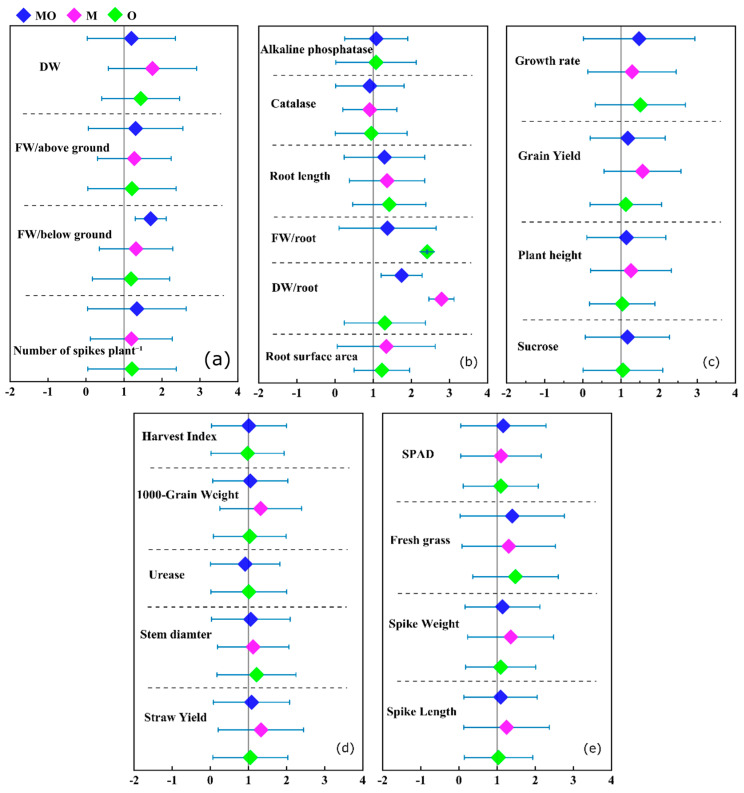
Effects of organic fertilizer (O), microbial fertilizer (M), and combined application of organic fertilizer and microbial fertilizer (MO) on the plant traits (**a**). morphological characteristics; (**b**). plant biomass (FW: fresh weight, DW: dry weight); (**c**). Physiological and nutrient indices; (**d**). growth indices; (**e**). yield parameters.

Under saline–alkali stress, the application of organic fertilizer, microbial inoculant, and their combination increased plant dry weight and spike numbers. Among them, the combined application significantly increased aboveground dry weight (Figure 2a). Microbial inoculant exhibited the most prominent promoting effect on root biomass (fresh and dry weight), while organic fertilizer and the combined treatment showed no significant effects on root morphological indices (Figure 2b). The crop growth indices, including growth rate, yield, plant height, and sucrose activity were increased a litte but not significantly, probably limited by experimental conditions or sample size (Figure 2c). Similarly, the amendments generally exerted positive impacts on indices including harvest index, 1000-grain weight, urease activity, stem diameter, and straw yield, although the effects were not significant (Figure 2d). Under saline–alkali conditions, organic fertilizer, microbial inoculant, and the combined application potentially promoted crop photosynthetic performance, fresh forage yield, and spike development, but these enhancing effects lacked statistical stability (Figure 2e).

Overall, the combined application of organic fertilizer and microbial inoculant represents the optimal strategy for the improvement of saline–alkali soil–crop systems. Its advantages are reflected in significantly enhancing soil nutrient retention capacity, activating phosphorus-cycling enzyme activities, optimizing ion composition, and indirectly improving the crop growth environment.

In practice, microbial agents cannot only act directly on plants but also reshape soil microbial community structure and function in the long run, increasing microbial diversity and beneficial bacterial abundance while shifting microbial interactions from competition to cooperation [2,7,10,17,18,19,21,23,24], a phenomenon mirrored by the enhanced microbial network complexity observed in halophyte rhizospheres [25]. Such community structural optimization activates soil ecosystem vitality, significantly enhances key soil enzyme activities, and enriches functional genes related to nitrogen fixation, nitrification, and phosphate mineralization [17,21]. Ultimately, it improves soil organic matter content, reduces salinity and alkalinity, enhances aggregate stability, and comprehensively promotes soil health [2,10,17,23].

**Table 1:** Functional microorganisms, main mechanisms and experimental forms for microbial remediation in saline−alkali soil.

Study/Application Stage	Main Research Content	Functional Microorganisms (Species/Combinations)	Main Mechanisms/Functions	Soil Type	Experimental Form	References
Single-function strains	Physiological mechanism of ACC deaminase-producing plant endophytic bacteria alleviating salt stress in wheat	*Kocuria rhizophila, Cronobacter sakazakii*	Produce ACC deaminase to reduce stress ethylene. Producing IAA, solubilizing phosphorus, and inducing plant antioxidant systems.	80 mM and 160 mM NaCl stress for simulation, no soil pH/EC reported.	Greenhouse control experiments	[26]
Single-function strains	Screening and identifying highly effective salt-tolerant growth-promoting bacteria from saline soil to assess their growth-promoting potential for rice seedlings	*Brevibacterium sediminis*	Extremely high salt tolerance (12% NaCl). Potentially producing extracellular polysaccharides, ACC deaminase, IAA, etc.	coastal salt-affected areas, no soil pH/EC reported.	Indoor Petri dish tests	[12]
Single-function strains	Screening, multifunctional characterization and genome-wide analysis of the multifunctional strain *Bacillus velezensis*	*Bacillus velezensis*	Possesses multiple functions including biological control, plant growth promotion, and saline–alkali tolerance.	strain tolerates 10% NaCl and pH 9.5 in vitro; no soil pH/EC reported.	In vitro and in pot experiments	[13]
Synthetic microbial communities	Construction of functionally complementary complex microbial agents and exploration of their remediation mechanisms	*B. oceanisediminis (phosphorus solubilization) + A. indicus (nitrogen fixation)*	Nutritional complementarity and metabolic coupling (phosphorus-nitrogen exchange) between strains, synergistic effect.	initial pH/EC not reported	Pot experiment	[17]
Synthetic microbial communities	Development of salt-tolerant microbial communities to mitigate alkaline soil stress	*L. fusiformis + L. sphaericus + B. licheniformis*	Possesses multiple extracellular hydrolase activities, ACC deaminase, IAA synthesis capacity and Na^+^ adsorption capacity.	sodic soil: pH > 8.5, exchangeable sodium percentage > 15%	Field trials	[27]
Synthetic microbial communities	Exploration of the impact of bacterial social interactions on rhizosphere community assembly and rational design of synthetic microbiota	Using *B. velezensis* as a model to design microbiota with different phylogenetic correlations	Social compatibility (such as group swimming integration) drives community assembly.	pH ≈ 6.8–7.0	Hydroponic and pot experiments	[6]
Synthetic microbial communities	Evaluation of the growth-promoting effects of salt-tolerant bacteria alone and in combination on wheat under salt stress	*B. velezensis*, *C. thuringiensis frigoritolerans*	Three strains functionally complementary (covering IAA, ACC deaminase, phosphorus solubilization, nitrogen fixation, and iron carrier).	50/100/200/300/400 mM NaCl	Potd experiment	[9]
Synthetic microbial communities	Construction of synthetic microbiota of salt-tolerant phosphorus-solubilizing bacteria and effect evaluation	*Kluyvera* sp. *+ Klebsiella* sp./*Enterobacter* sp. *+ Klebsiella* sp.	The combination of the two bacteria showed synergistic enhancement in phosphorus solubilization, IAA production, and antioxidant enzyme activity.	1% (*w*/*v*) NaCl to simulate saline conditions	Pot experiment	[7]
Bactericate-improver synergy	Synergistic remediation of rice systems by combination of bacteria and unsterilized organic fertilizer	*Bacillus* sp., *Aspergillus* sp., *Penicillium* sp.	Synergistically boost enzyme activity and reshape the microbial community. Indigenous microorganisms in UOF form a “microbial scaffold”.	saline–alkali soil amended with 1.0% or 1.5% Na_2_CO_3_ (*w*/*v*) to simulate sodic-saline stress	Pot experiments	[18]
Bactericate-improver synergy	Functionally complementary salt-tolerant microbiota combined with organic amendments	SynCom (*B. velezensis* + *B. marisflavi*) + biochar (22.5 t·ha^−1^) + sheep manure (7.5 t·ha^−1^)	Combined organic modifiers create a favorable microenvironment and reshape the microbiota interaction network.	saline–alkali soil (pH 8.13, EC 1.83 dS/m, SOM 7.36 g·kg^−1^)	Potted experiment	[19]
Inoculant-improver	Field application of soil conditioners based on salt-tolerant synthetic microbial communities to hybrid rice	*B. subtilis + B. licheniformis + Streptomyces* spp.	Microbial communities compounded with organic/inorganic carriers. Enhance plant antioxidant and photosynthetic product transport.	paddy soil irrigated with freshwater–seawater mix (EC 11 dS/m) to simulate high-saline conditions	Field trials	[20]
Inoculant–amendment synergy	Mechanism of soil organic carbon sequestration by combination of organic amendments and microbial agents	Compound microbial agent (*B. subtilis* et al.) + organic conditioner (cow dung + humic acid)	Synergistic treatment drives the transformation of soil bacterial communities, enhancing carbon utilization efficiency.	saline–alkali soil, initial soil pH/EC not reported	Two-year field trial	[21]

The efficiency of microbial remediation in saline–alkali soils is determined by soil texture, organic matter, and nutrient status. Sandy soils promote PGPR colonization by 15–20%, whereas clay soils hinder metabolite diffusion and weaken plant–microbe interactions [17,23]. Low organic carbon severely reduces microbial survival, with inoculant survival rates below 5% in soils with less than 1% organic matter, requiring microbial agents to be combined with organic amendments [18,21]. Nutrient availability is equally critical. When available phosphorus falls below 5 mg/kg, the growth and function of nitrogen-fixing and phosphorus-solubilizing microbes are significantly inhibited [15,28]. The positive feedback between microbial colonization and soil nutrient improvement only occurs under adequate initial nutrient conditions.

The differences in remediation effects of different application strategies essentially stem from the functional diversity of these microorganisms and their synergistic pathways. The following section synthesizes core microbial resources and their multi-dimensional mechanisms, establishing a unified “structure–function–mechanism” framework to elucidate the logic of microbial-mediated stress alleviation.

## 3. Microbial Functions

Although saline–alkali soils have generally lower microbial diversity than that of non-saline soils, they harbor a large number of specialized stress-adapted microorganisms with strong environmental tolerance. These are unique microbial resources for developing saline–alkali soil remediation agents [3,29]. Researchers isolated and identified core functional microbial taxa with remediation potential from extreme habitats, such as saline soils and the rhizospheres of halophytes. These include salt-tolerant plant growth-promoting rhizobacteria (PGPR), such as *Bacillus* [15], *Pseudomonas* [30], and *Halomonas* [31], alongside other genera with confirmed remediation efficacy [24,32]. Furthermore, dark septate endophytes (DSE) form mutualistic symbioses with plant roots, expanding the absorptive surface area for water and nutrients [33,34]. Phosphorus-solubilizing microorganisms activate fixed phosphorus in the soil [28], while endophytic fungi colonize plant roots to enhance host resilience [16,35,36]. Certain alkaliphilic taxa thrive in high-pH environments and are specifically suited for soda saline–alkali soil remediation [37].

Within the plant–soil microecosystem, microbes drive synergistic effects via direct and indirect pathways. These comprise five interrelated categories.

- Ion Concentration Regulation and Osmotic Stabilization

Ion transport regulation: Microbes secrete signaling molecules (e.g., indole-3-acetic acid and extracellular peptides) to activate the plant plasma membrane-localized SOS1 (Salt Overly Sensitive) pathway. Through phosphorylation cascades, they upregulate the expression of plasma membrane Na^+^/H^+^ antiporters (SOS1) and tonoplast Na^+^/H^+^ antiporters (NHX1), while enhancing inward-rectifying K^+^ channel (AKT1) activity. This promotes Na^+^ efflux and K^+^ retention, maintaining intracellular K^+^/Na^+^ homeostasis [38,39,40]. Some halotolerant microbes possess genes encoding Na^+^/H^+^ antiporters, such as *nhaA* (mediating Na^+^ efflux) and *mrp* (sequestering Na^+^ intracellularly for osmotic balance), thereby modulating rhizosphere Na^+^ activity and reducing plant-available Na^+^ [37,41].

Osmoprotectant synthesis: Microbes activate operons including *otsAB* (trehalose synthesis) and *betIBA* (glycine betaine synthesis) to produce compatible solutes. These small molecules are transported to plant root cells, lowering cellular osmotic potential and preventing dehydration [14,29].

EPS-mediated protection: Microbes synthesize extracellular polymeric substances (EPSs) via glycosyltransferases encoded by the EPS gene cluster. Anionic groups (e.g., carboxyl and hydroxyl) on EPS bind free Na^+^ via electrostatic interactions, limiting Na^+^ influx into roots. Additionally, EPS binds soil particles, reducing bulk density, increasing porosity, and improving water-holding capacity [31,42].

- Hormone and Ethylene Regulation

Ethylene modulation: Microbes express ACC deaminase (encoded by *acdS*), which hydrolyzes ACC (the precursor of ethylene) into α-ketobutyrate and ammonia. This reduces stress-induced ethylene synthesis, preventing the inhibition of root elongation and cell division [4,14,29,30,39,43]. Some microbes also regulate ethylene signaling genes (e.g., *CTR1*, *EIN2*) to attenuate transduction.

Growth promotion: Diverse microbes synthesize phytohormones such as auxins, cytokinins, and gibberellins. These directly stimulate root meristem activity, promoting lateral root and root hair development to expand the absorptive area [11,30,44].

- Antioxidant Response Activation

Microbes enhance plant reactive oxygen species (ROS) scavenging capacity via systemic signal transduction. Microbe-associated molecular patterns (e.g., lipopolysaccharides, peptidoglycans and flagellin) are recognized by pattern recognition receptors on the plant root cell membrane, triggering downstream *MAPK* cascade phosphorylation reactions. This process activates the expression of transcription factors related to the plant antioxidant system, upregulates the transcription levels of genes encoding antioxidant enzymes, including superoxide dismutase (SOD), catalase (CAT), and peroxidase (POD), and increases enzyme activity. As a result, the decomposition of ROS such as superoxide anions and hydrogen peroxide is accelerated, and the accumulation of malondialdehyde (MDA), a product of membrane lipid peroxidation, is reduced, thus alleviating damage to the plant cell membrane system [10,16,27,36,44].

- Key Nutrient Cycle Activation

Microbes secrete functional enzymes to alleviate nutrient limitation under saline–alkali stress.

Nitrogen fixation: Diazotrophs carrying the *nifHDK* gene cluster reduce atmospheric N_2_ to ammonium under microaerobic conditions. Salt-tolerant nitrogenase variants or osmoprotectant accumulation allow these microbes to maintain activity in saline–alkali environments [15,29].

Phosphorus solubilization: Microbes secrete organic acids (e.g., citric and oxalic acid via TCA cycle upregulation) and enzymes (phytase and phosphatase), converting insoluble phosphates into bioavailable forms and alleviating pH-induced P-fixation [34].

Iron mobilization: Microbes synthesize high-affinity siderophores via the sidgene cluster, chelating Fe^3+^ into soluble complexes absorbable via root transporters, counteracting iron deficiency chlorosis common in high-pH soils [29].

- Systemic Resistance Induction

Microbes activate plant defense signaling pathways to improve comprehensive stress resistance: Microbes and their metabolites (e.g., oligosaccharides, volatile organic compounds, and secondary metabolites) act as elicitors that activate salicylic acid (SA)- and jasmonic acid (JA)/ethylene (ET)-mediated plant defense signaling pathways. This upregulates pathogenesis-related (PR) proteins and phytoalexins, promoting lignin and callose deposition in cell walls. These responses enhance tolerance to saline–alkali stress and improve resistance to biotic challenges like pathogens [11,31,39].

**Figure 3:**
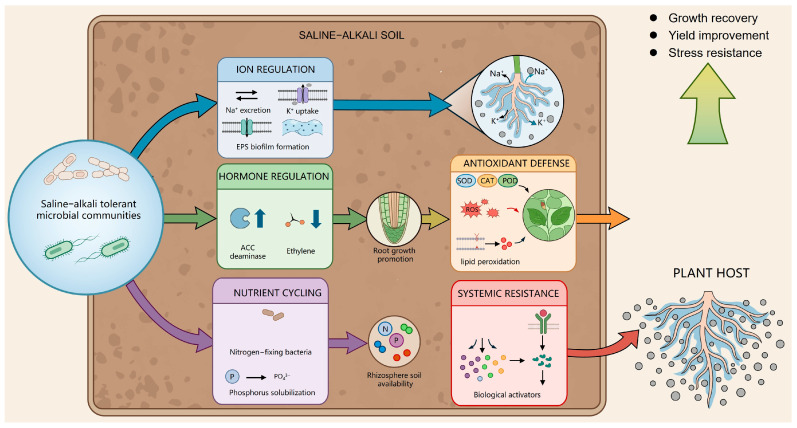
Mechanism of plant stress resistance and growth recovery mediated by the pro-biota of salt-tolerant plants under salt stress.

This review establishes a “structure–function–mechanism” framework for microbial communities in saline–alkali soils (Figure 3). This framework integrates disparate microbial resources and functional pathways into a cohesive network. For instance, when constructing SynComs, researchers can select strains with specific attributes corresponding to the five modules—such as haloalkaliphiles with nhaAactivity [37], organic acid-secreting P-solubilizers [34], or ACC deaminase producers [4,29]—to ensure functional complementarity.

It should be noted that microbial consortia exhibit emergent properties unpredictable from single-strain functions. Dynamic feedback loops exist between community assembly and the environment. Exogenous inoculation reshapes native networks, which in turn regulate colonization efficiency and functional expression, a process not captured by static module matching [19,24]. Notably, microbial volatile organic compounds (VOCs) serve as long-distance signals that simultaneously trigger multiple modules. For instance, 2,3-butanediol and acetoin from *Bacillus* can upregulate plant *SOS1* expression, induce JA/ET-dependent systemic resistance, and promote auxin synthesis to optimize root architecture, thereby synergistically improving salt tolerance [45].

Having established these fundamental pathways of microorganisms, and on this basis, recent studies have further expanded the application scenarios. The following section expands upon frontier progress in microbial remediation technology through cross-disciplinary integration. The following section introduces the frontier progress in this field, including new microbial and novel interaction forms and cross-technology integration paradigms.

## 4. Restoration Mechanisms and Frontier Applications

In addition to direct contact, microorganisms communicate over long distances through volatile organic compounds. Bacterial VOCs not only directly induce plant salt tolerance but also inhibit soil-borne pathogens and reshape the rhizosphere microbiota interaction network, important for regulating soil health [45].

Synthetic microalgae–microbe symbiotic partnership is emerging as a promising remediation strategy for open saline–alkali land, especially for saline soils with low organic matter content. Phototrophic salt-tolerant microalgae fix CO_2_ and excrete dissolved organic carbon (e.g., simple sugars and organic acids) and O_2_, thereby fueling heterotrophic beneficial bacteria and fungi. In return, heterotrophic microbes mineralize organic nutrients, produce growth-promoting hormones, and release CO_2_ through respiration to support microalgal photosynthesis. This self-sustaining resource circulation greatly reduces the dependence of the remediation system on external nutrient inputs [46]. Simultaneously, the consortium achieves multifaceted remediation: algal extracellular polysaccharides adsorb Na^+^ and promote soil aggregation, while heterotrophs solubilize phosphorus and fix nitrogen. The metabolic division of labor confers higher stress resilience than single-taxon inoculants, maintaining functionality under high salinity and drought [46].

The rhizosphere and rhizoplane are hotspots for plant–microbe interactions. Research shows that salt-tolerant plants do not passively endure saline–alkali stress, but actively recruit specific salt-tolerant microbial communities to colonize these two niches, forming a functional protective barrier around the root system [33,47]. This adaptive host strategy represents a novel target for rhizosphere engineering, wherein manipulating the root-associated microbiome enhances plant resilience to abiotic stress [33,47]. Complementing this biological approach, nanomaterials (e.g., silicon nanoparticles) serve as carriers to facilitate the colonization of beneficial microbes or act as physical barriers against sodium influx, thereby synergizing with bio-inoculants to mitigate stress [22]. Furthermore, host-guided multi-generational selection, analogous to conventional crop breeding, enables the rapid development of robust, functional salt-tolerant consortia [7]. It should be noted that this effect was observed under controlled conditions, and the yield increase rate decreased to 15–20% in field due to the interference of native microbial communities [7].

Meanwhile, several research groups and companies are developing artificial intelligence (AI)-assisted approaches to design microbial consortia for saline–alkali soil remediation. At present, this is still at the stage of laboratory verification and small-scale pilot testing. Machine learning models have been used to predict the compatibility of functional strains and the remediation effects under specific soil conditions, showing higher screening efficiency than traditional methods. However, there is a lack of large-scale application of AI-designed microbial products in saline–alkali soils [48,49]. This technology is expected to become an important tool for the development of next-generation microbial remediation agents field adaptation verification and data model generalization are further improved.

While these frontier studies highlight immense potential, significant bottlenecks impede large-scale deployment. The following section analyzes the key constraints—including field instability, product development hurdles, and safety assessment gaps—and proposes future directions to bridge the lab-to-field gap.

## 5. Prospect of Large-Scale Application

Microbial agents have demonstrated considerable potential for rehabilitating saline–alkali soils. However, transitioning from lab-to-field application is hindered by significant challenges. The most prominent issue is the instability of field efficacy; while robust growth promotion is often observed in vitro, these effects frequently diminish under complex natural conditions due to variations in soil type, climate, tillage practices, and intense competition from indigenous microbial communities [4,11,14,15,50,51].

### 5.1. The Laboratory–Field Efficacy Gap: Mechanisms and Evidence

A critical barrier to scalability is the discrepancy between lab-scale results and field outcomes. Current experimental methodologies often introduce biases that overestimate practical viability. Most laboratory studies utilize sterilized substrates, eliminating the competitive pressure and protistan grazing inherent in natural soils, leading to colonization rates that are 2–3 times higher than those observed in fields [8,10]. Furthermore, static lab conditions fail to replicate the dynamic fluctuations of water and salt driven by rainfall or irrigation. For instance, while *Bacillus*-based consortia increased rice biomass by 42% in pot experiments [10], field trials in Jiangsu coastal areas yielded only a 12–18% increase due to tidal immersion and intermittent anoxia disrupting rhizosphere stability [20].

This gap is further evidenced by specific case studies:

Microbial Competition: In the Hetao Irrigation District, *Halomonas inoculants* reduced wheat leaf Na^+^ by 35% in the lab [30], but field efficacy dropped to <10% [21]. This is attributed to the stable cooperative network of native microbiota, which restricts exogenous Halomonasto < 3% of the rhizosphere niche (compared to ~25% in sterile systems).

Abiotic Stress Interference: Under 150 mM salt stress, mixed consortia of phosphate-solubilizing bacteria and dark septate endophytes (DSE) boosted maize biomass by 38% [16]. Yet, in the hyper-arid saline areas of Northwest China (<200 mm precipitation), yield increases were merely 8% [11]. Severe field drought reduced root exudation by ~70%, severing the chemical signaling required to induce plant systemic resistance.

### 5.2. Core Bottlenecks Limiting Scalability

Beyond ecological interactions, three interrelated bottlenecks constrain large-scale adoption:

1. Technical Limitations in Product Development.

For compound microbial agents, high-throughput compatibility screening remains scare. Traditional co-culture methods verify fewer than 10 strain combinations daily, failing to meet the demands of screening extensive functional libraries [6,9]. Incompatible strain combinations lead to competitive inhibition during storage and application, potentially slashing remediation efficacy by 50–70% [8,9]. Additionally, formulation processes (carrier selection, embedding technology) and shelf-life stability require further optimization to ensure rhizosphere colonization post-application [4,14,31].

2. Unquantified Long-term Ecological Risks.

Current safety assessments are predominantly short-term (<1 year). There is a paucity of data regarding the persistence of exogenous strains (which can be detected three years post-inoculation) and their impact on the native microbial food web, including protists and nematodes [3,52]. Moreover, the potential for horizontal gene transfer between engineered strains and indigenous microbiomes raises concerns about unintended shifts in ecosystem function [4].

3. Lack of Standardized Application Guidelines.

Efficacy is highly dependent on the matching of strains with specific soil types and crop varieties. Currently, there is no unified technical specification for inoculation doses or timing across diverse agro-ecological zones, contributing to the instability of field results [11,15,20].

### 5.3. Future Pathways: From Smart Design to Implementation

To surmount these obstacles, future research must integrate advanced technologies and holistic management strategies.

Mechanistic Insights: Leveraging multi-omics approaches (genomics, transcriptomics, metabolomics) is essential to decipher the complex chemical dialogs (quorum sensing, volatile organic compounds) within plant–microbe-environment systems [3,32,39,40,53]. Attention should extend to understudied taxa like archaea and viruses to fully understand ecosystem dynamics.

Next-Generation Products: Artificial intelligence and machine learning can accelerate the design of synthetic consortia by predicting optimal strain combinations [5,6,7]. Synthetic biology tools, such as CRISPR, offer avenues to engineer enhanced traits, including improved salt tolerance and root colonization efficiency [5,37]. Furthermore, novel delivery systems utilizing nanomaterials or natural polymers could enable precise, targeted release of microbial agents [5,22].

Regional Adaptation and Policy: Large-scale application requires establishing localized guidelines through long-term, standardized field trials across diverse global ecoregions. Notably, current research is heavily biased toward the East Asian, South Asian, and European temperate zones [6,8,10,15,20,21,29]. Systematic studies in the African Sahel, the American Great Plains, and high-latitude cold regions are urgently needed to validate the universality of current conclusions. Ultimately, microbial remediation should be integrated into conservation agriculture frameworks, working synergistically with stress-tolerant cultivars and water-saving irrigation to build climate−resilient farming systems [1,53,54].

### 5.4. Socio-Economic Value and Conclusion

While traditional physicochemical remediation methods are often cost-prohibitive and environmentally taxing, microbial bioremediation offers a sustainable alternative that enhances soil health and crop productivity [55,56]. The technology presents significant economic value by boosting yields (e.g., >20% in rice with organic amendments [20]) and fostering new bio-industry sectors [57]. However, the core challenge remains bridging the gap between microbial potential and tangible farmer profitability. Future efforts must focus on demonstrating not just agronomic benefits, but also ecosystem service values—such as carbon sequestration and biodiversity conservation—to drive policy support and widespread adoption in the global fight against soil salinization [3,21,58].

It should be noted that most studies cited herein are concentrated in three typical saline–alkali regions: the East Asian monsoon region (coastal and inland soda saline soils in China [10,20,21]), the South Asian arid-semiarid region (saline farmlands in India and Pakistan [15,29]), and the European temperate region (Mediterranean salt-affected soils [6,8]). Consequently, there is a paucity of systematic studies on ecosystems in the African Sahel, the American Great Plains, and high-latitude cold regions, which limits the universal applicability of current conclusions. The functional strains and strategies summarized here are primarily applicable to these well-studied regions; targeted screening of local native microbial resources remains essential for other saline–alkali environments to achieve stable remediation effects. Furthermore, while existing research predominantly focuses on agronomic benefits (yield increase, soil property improvement), perspectives on ecosystem services—such as biodiversity conservation and hydrological regulation—remain scarce and represent a vital direction for future research [3,21].

## 6. Conclusions

In summary, this review synthesizes current knowledge regarding microbial remediation of saline–alkali soils. The field has shifted from the use of single, highly effective bacterial strains to the rational design of synthetic microbial communities (SynComs) that operate through ecological complementarity. This review summarizes five mechanisms by which beneficial microbes support plants under saline–alkali stress: regulation of ion and osmotic balance, modulation of plant hormones, activation of antioxidant responses, mobilization of key nutrients, and induction of systemic resistance. Looking ahead, tools such as artificial intelligence and genetic engineering are expected to help develop more efficient microbial products. However, several major challenges must be addressed before this technology can be widely applied in agricultural systems. Field performance remains inconsistent and common standards for product quality and safety assessment are still lacking. Future work should focus on elucidating the complex connections between plants, microbes, and the environment, and on developing reliable products suited to climate-smart farming systems. The ultimate goal is to integrate robust, eco-friendly microbial solutions into climate-resilient farming systems, offering a sustainable pathway for global soil restoration.

## Data Availability

No new data were created or analyzed in this study.

## Supplementary Materials

The following supporting information can be downloaded at: https://www.mdpi.com/article/10.3390/life16060938/s1, Data processing procedures for Figure 1 and Figure 2.
