# Supplementary material for: From Single Strains to Synthetic Bacterial Communities: Microbial Remediation in Saline–A-Alkali Soil
Source: Life (Basel). 2026 Jun 2;16(6):938. doi: 10.3390/life16060938 (PMC13302735; doi:10.3390/life16060938)
Supplement: Supplementary file 1 [file life-16-00938-s001.zip › life-4262500-supplementary.pdf]

Supplementary

From Single Strains to Synthetic Bacterial Communities: Microbial Remediation in Saline-alkali Soil

Juanjuan Wang [1,2], Wen Huang [1], Jiaying Cai [1], Hengjia Zhang[1], Xiaoqing Qian[1,2*]

[1] Key Laboratory of Arable Land Quality Monitoring and Evaluation (Yangzhou University), Ministry of Agriculture and Rural Affairs, Yangzhou 225127, China; wangjuanjuan@yzu.edu.cn

[2] Jiangsu Collaborative Innovation Centre for Solid Organic Waste Resource Utilization, Nanjing, Jiangsu 210095, China

*Correspondence:qianxq@yzu.edu.cn

Data processing procedures for Figure 1 and Figure 2

To quantify the relative percentage changes in the physicochemical properties of saline-alkali soil and plant growth parameters resulting from the application of organic fertilizer and microbial inoculants, this study used the natural logarithm of the response ratio (R) as the effect value to measure the degree of response of soil and plant indicators to the application of organic fertilizer and microbial inoculants. First, the natural logarithm of the response ratio (lnR) was calculated using Equation (1):

$\ln R = \ln (X_t / X_c)$(1)

Where X_t and X_c represent the measured values of the selected dependent variables for the treatment groups (organic fertilizer, microbial inoculants, and their combined application) and the control group, respectively. In the database, experiments involving different types of soil amendments used the absence of any amendment as the control; experiments involving different types of microbial inoculants used the absence of inoculation as the control; and trials involving different application rates used the untreated condition as the baseline control.Subsequently, the weighted average effect value for all observed values was calculated using Equation (2):

$EF = \exp [\Sigma \ln R(i) \times W(i) / \Sigma W(i)]$(2)

Where W(i) is the weight of the observed value for the *i*th study.EF is the weighted effect value, expressed as a relative change factor; a value greater than 1 indicates an increase relative to the control group, while a value less than 1 indicates a decrease.

In the meta-analysis, the variables from each study were categorized into the following functional groups: soil physicochemical properties, electrical conductivity (EC), pH, soil organic carbon (SOC), salt content, total nitrogen (TN), total organic carbon (TOC), exchangeable sodium percentage (ESP), cation exchange capacity (CEC), water-soluble sodium (WSNa), water-soluble potassium (WSK), water-soluble magnesium (WSMg), water-soluble calcium (WSCa), available potassium (AK), available phosphorus (AP), alkali-hydrolyzable nitrogen (AN), sulfate (SO_4^{2-}), bicarbonate (HCO_3^-), β-glucosidase activity, and alkaline phosphatase activity, as well as plant growth parameters, including dry weight (DW), fresh weight of aboveground parts (FW/above ground), fresh weight of belowground parts (FW/below ground), number of spikes per plant (Number of spikes plant-1), alkaline phosphatase activity (Alkaline phosphatase), catalase activity (Catalase), root length (Root

length), fresh weight of roots (FW/root), root dry weight (DW/root), root surface area, growth rate, grain yield, plant height, sucrose content, harvest index, 1000-grain weight, urease activity, stem diameter, straw yield, SPAD value (SPAD, relative chlorophyll content), fresh grass yield, spike weight, spike length. Forest plots were generated using OriginPro 2024.

References of Data Sources

[1] Abbas H M M, Li S, Zhou W, Sultan H, Khan M N, Shah A, Tahir A, Iltaf H, Mu Y, Nie L . Improving Rice Root Development and Soil Health in Saline Soils: A Biochar and Microbial-Inoculated Biochar with Nitrogen Approach . Plants . 2026, 15 (6) .

[2] Abd El-Tawwab A R, Abdelkhalik A, Shaaban A, Abdel-Moatamed B R, Hemida K A, Sayed A A S, Rady M M, Saudy H S, Gyushi M A H .Compost-mixed Biochar Improves Saline Soil Properties and Enhances Brassica Oleracea var. Capitata physio-biochemical Responses . Journal of Soil Science and Plant Nutrition . 2026, 26 (1): 1665-1681 .

[3] Alotaibi M M, Aljuaid A, Alsudays I M, Aloufi A S, AlBalawi A N, Alasmari A, Alghanem S M S, Albalawi B F, Alwutayd K M, Gharib H S, Awad-Allah M M A . Effect of Bio-Fertilizer Application on Agronomic Traits, Yield, and Nutrient Uptake of Barley (Hordeum vulgare) in Saline Soil . Plants . 2024, 13 (7) .

[4] Bai L, Zhang R, Wu S, Liu B, Li Y, Wang X, Zhao B . Comparative Effects of Biochar and Humic Acid on the Soil–Wheat System in Mildly Saline Soils . Agronomy . 2026, 16 (5) .

[5] Bai X, Wu J, Zhang B, Zhao H, Tian F, Wang B . Metagenomics reveals functional profiles of soil nitrogen and phosphorus cycling under different amendments in saline-alkali soil . Environmental Research . 2025, 267 .

[6] Bian Q, Yang Y, Ma L, Ma Y, Wang Z, Fu Y, Feng Y, Zhu J, Wei Y, Lin L . Effects of combined technology of micro-/nanobubble oxygenation irrigation and microbial agents on saline soil remediation and cotton yield . Frontiers in Plant Science . 2025, 16 .

[7] Cai Y, Ren L, Wu L, Li J, Yang S, Song X, Li X . Saline-alkali soil amended with biochar derived from maricultural-solid-waste: Ameliorative effect and mechanism . Journal of Environmental Management . 2024, 368 .

[8] Che N, Qu J, Wang J, Liu N, Li C, Liu Y . Adsorption of phosphate onto agricultural waste biochars with ferrite/manganese modified-ball-milled treatment and its reuse in saline soil . Science of The Total Environment . 2024, 915 .

[9] Chen M, Zhang S, Liu L, Wu L, Ding X . Combined organic amendments and mineral fertilizer application increase rice yield by improving soil structure, P availability and root growth in saline-alkaline soil . Soil and Tillage Research . 2021, 212 .

[10] Chen Z, Li Y, Hu M, Xiong Y, Huang Q, Jin S, Huang G . Lignite bioorganic fertilizer enhanced microbial co-occurrence network stability and plant–microbe interactions in saline-sodic soil . Science of The Total Environment . 2023, 879 .

[11] Ding H, Qin H, Liu M, Wang C . New Type of Superabsorbent Polymer Reinforced with Vermicompost and Biochar to Enhance Salt Tolerance of Sesbania cannabina in Severely Saline-Alkali Soils . Agronomy . 2026, 16 (2) .

[12] El-Egami H M, Hegab R H, Montaser H, El-Hawary M M, Hasanuzzaman M . Impact

of Potassium-Solubilizing Microorganisms with Potassium Sources on the Growth, Physiology, and Productivity of Wheat Crop under Salt-Affected Soil Conditions . Agronomy . 2024, 14 (3) .
[13] Fan L, Zhang P, Cao F, Liu X, Ji M, Xie M . Effects of AMF on Maize Yield and Soil Microbial Community in Sandy and Saline Soils . Plants . 2024, 13 (15) .
[14] Han L, Li Y, Ma Z, Li B, Liang Y, Gao P, Zhao X . Alleviation of Saline–Alkaline Stress in Alfalfa by a Consortium of Plant-Growth-Promoting Rhizobacteria . Plants . 2025, 14 (17) .
[15] Huang L, Wang Z, Chen L, Feng Y, Zhang F, Liu J, Zhang C, Wang L, Chen L, Guo F . Synergistic improvement of saline-alkali soil quality by desulfurized gypsum and Sesbania cannabina intercropping: Microbial mechanisms and field efficacy . Plant and Soil . 2026, 520 (2): 1211-1229 .
[16] Huo Q, Gong M, Jiang Y, Yang X, Kong M, He J, Zhang Q, Song J, Li X, Han W, Mei X, Lv G . Microencapsulated Microbial Seed Coating Could Improve Soil Environment and Maize Grain Yield in Saline Soil . Plants . 2024, 13 (22) .
[17] Jin X, Liu X, Wang J, Chang J, Li C, Lu G . Rhizosphere Growth-Promoting Bacteria Enhance Oat Growth by Improving Microbial Stability and Soil Organic Matter in the Saline Soil of the Qaidam Basin . Plants . 2025, 14 (13) .
[18] Lei S, Gong H, Li J, Xu Y . Reconsidering "4 per 1000" target in mild salt-affected lands: A case study on exogenous carbon inputs . Farming System . 2026, 4 (3) .
[19] Li D, Qi Z, Guo J, Wang T, Li X, Hou N . Study on the screening of high-efficiency salt and alkali-tolerant microbial agents and their roles and mechanisms in enhancing saline-alkaline soil remediation . Journal of Cleaner Production . 2025, 519 .
[20] Li G, Shan Y, Nie W, Sun Y, Su L, Mu W, Qu Z, Yang T . Humic acid improves water retention, maize growth, water use efficiency and economic benefits in coastal saline-alkali soils . Agricultural Water Management . 2025, 309 .
[21] Li N, Sheng K, Zheng Q, Hu D, Zhang L, Wang J, Zhang W .Inoculation with phosphate-solubilizing bacteria alters microbial community and activates soil phosphorus supply to promote maize growth . Land Degradation & Development . 2022, 34 (3): 777-788 .
[22] Li S, Yin H, Ren X, Li X, Ren S, Zhang M ."Biochar-microbe interaction promotes soybean growth in Saline-alkali land by improving soil nutrients and regulating the rhizosphere microbial Community" . World Journal of Microbiology and Biotechnology . 2026, 42 (2) .
[23] Li Y, Zhang J, Wang X, Feng Z, Yang E, Wu M, Jiang Y, Huang J, Gao Z, Du Y . The synergistic effect of extracellular polysaccharide-producing salt-tolerant bacteria and biochar promotes grape growth under saline-alkaline stress . Environmental Technology & Innovation . 2025, 38 .
[24] Liu J, Ji Y, Xie W, Yang J, Wang X, Feng Y, Yu X, Yao R, Yang J R, Zhu H . Synergistic suppression of ammonia volatilization by biochar–compost through soil physicochemical and microbial regulation in saline–alkali soil . Journal of Environmental Management . 2026, 405 .
[25] Liu J, Shi Z, Zhang L, Feng R, Zhang G, Zou H, Wang G, Yang Y . Contrasting acidic and alkaline biochar reprogram alfalfa metabolism and rhizosphere microbiomes in

saline-alkali soils．Biochar．2026，8（1）．
[26] Liu J，Zhu Y，Wu H，Dong G，Zhou G，Smith D L．Effects of Fertilizers and Soil Amendments on Soil Physicochemical Properties and Carbon Sequestration of Oat (Avena sativa L.) Planted in Saline–Alkaline Land．Agronomy．2025，15（7）．
[27] Liu T，Wang H，Jia Y，Sun H，Li M，Chen W，Liu T．Effects of High-Rate Organic Amendments Combined with Supporting Management Practices on Topsoil Amelioration and Yield Improvement in Coastal Saline–Alkali Farmland．Water．2026，18（6）．
[28] Luo D，Shi J，Li M，Chen J，Wang T，Zhang Q，Yang L，Zhu N，Wang Y．Consortium of Phosphorus-Solubilizing Bacteria Promotes Maize Growth and Changes the Microbial Community Composition of Rhizosphere Soil．Agronomy．2024，14（7）．
[29] Luo X，Liu G，Xia Y，Chen L，Jiang Z，Zheng H，Wang Z．Use of biochar-compost to improve properties and productivity of the degraded coastal soil in the Yellow River Delta, China．Journal of Soils and Sediments．2016，17（3）：780-789．
[30] Ma L，Li Y，Zhang Y，Li Y，Wei J，Liu Z，Tan D．Synergistic Application of Humic Acid and Microbial Fertilizers Improve Soil Quality, Reshape Microbial Network, and Enhance Wheat Yield in Coastal Saline–Alkali Soils．Microorganisms．2025，13（12）．
[31] Masrahi A S，Alasmari A，Shahin M G，Qumsani A T，Oraby H F，Awad-Allah M M A．Role of Arbuscular Mycorrhizal Fungi and Phosphate Solubilizing Bacteria in Improving Yield, Yield Components, and Nutrients Uptake of Barley under Salinity Soil．Agriculture．2023，13（3）．
[32] Mei Y，Kayoumu M，He T，Cui X，Duan G．Enhancing salt tolerance and growth of Oryza sativa L. through biochar-bacteria composite amendment for potential application in coastal saline soil reclamation．Environmental Technology & Innovation．2026，41．
[33] Mishra P，Mishra J，Bharti C，Arora N K．Salt-Tolerant Pseudomonas taiwanensis PWR-1 Mediated Organic Acid Production for Biofortification of Zinc and Reducing Fertilizer Dependency in Wheat Under Saline Conditions．Journal of Plant Growth Regulation．2025，44（6）：3348-3368．
[34] Nawaz A，Shahbaz M，Asadullah，Imran A，Marghoob M U，Imtiaz M，Mubeen F．Potential of Salt Tolerant PGPR in Growth and Yield Augmentation of Wheat (Triticum aestivum L.) Under Saline Conditions．Frontiers in Microbiology．2020，11．
[35] Peng Y，Zhang H，Lv Z，Zhang J，Li G．Microbial inoculation improves soil aggregation by enhancing exopolysaccharides and lipopolysaccharides-related gene abundance in saline soil．Applied Soil Ecology．2025，214．
[36] Peng Z，Yang Q，Li X，Zhang X，Wang Z，Liang X，Xie J，Gao Z，Liu C．Interaction Between Nutrient-Laden Biochar and PGPR Reshapes Rhizosphere Microbiome to Reclaim Coastal Saline–Alkali Soil Fertility．Agriculture．2026，16（6）．
[37] Rahimi Chegeni A，Fatehi F，Ebrahimi A，Maleki M．Phosphate-Solubilizing Bacteria Modulated Salinity Stress in the Presence of Phosphorous through Improving Growth, Biochemical Properties, and Gene Expression of Chickpea (Cicer arientnum L.)．Journal of Soil Science and Plant Nutrition．2023，23（3）：4450-4462．
[38] Simarmata R，Khumairah F H，Erdayani E，Khairina Y，Christita M，Widowati T，

Lekatompessy S J R, Ngadiman . Synergistic Effects of PGPR and Reduced Fertilizer Application on Rhizosphere Diversity and Salinity Tolerance in Shallot (Allium cepa L. var. aggregatum) . Journal of Soil Science and Plant Nutrition . 2026, .

[39] Singh U B, Malviya D, Singh S, Singh P, Ghatak A, Imran M, Rai J P, Singh R K, Manna M C, Sharma A K, Saxena A K . Salt-Tolerant Compatible Microbial Inoculants Modulate Physio-Biochemical Responses Enhance Plant Growth, Zn Biofortification and Yield of Wheat Grown in Saline-Sodic Soil . International Journal of Environmental Research and Public Health . 2021, 18 (18) .

[40] Song J, Guan X, Chen L, Han Z, Cui H, Ma S . Cooperative Interplay Between PGPR and Trichoderma longibrachiatum Reprograms the Rhizosphere Microecology for Improved Saline Alkaline Stress Resilience in Rice Seedlings . Microorganisms . 2025, 13 (7) .

[41] Song X, Li H, Song J, Chen W, Shi L .Biochar/vermicompost promotes Hybrid Pennisetum plant growth and soil enzyme activity in saline soils . Plant Physiology and Biochemistry . 2022, 183: 96-110 .

[42] Sritongon N, Sarin P, Ketkaew S, Mongkolthanaruk W, Riddech N . Rice straw, biochar, cow manure as soil amendment and effective cellulolytic fungi immobilized in alginate biochar bead for enhancing soil enzyme activity and chemical characteristics of salt affected soil . Environmental Technology & Innovation . 2025, 40 .

[43] Suo F, Fu H, Liu J, Yin Z, Ma S, Dong L, Dong J, You X, Zhang L . Effect of individual and co-application of biochar and pyroligneous acid on corn seedling growth and microbial community of coastal soil . Environmental Technology & Innovation . 2025, 40 .

[44] Vatandoust M, Madandoust M, Rajaie M, Dejam M . Physiological and biochemical response of Echinacea purpurea to combined application of biofertilizers and salicylic acid under different irrigation regimes in saline conditions . Agricultural Water Management . 2026, 325 .

[45] Wang J, Muhammad R, Babar S, El-Desouki Z, Li Y, Wang X, Xia X, Jiang C . Insight into amelioration effect of iron-modified biochar on saline-alkali soil chemical properties and bacterial communities along a soil depth gradient . Pedosphere . 2025, 35 (5): 879-892 .

[46] Wang R, Liu T, Lu C, Zhang Z, Guo P, Jia B, Hao B, Wang Y, Guo W . Bioorganic fertilizers improve the adaptability and remediation efficiency of Puccinellia distans in multiple heavy metals-contaminated saline soil by regulating the soil microbial community . Journal of Hazardous Materials . 2023, 448 .

[47] Wang S, Li C, Wang X, Zhang C, Feng Y, Lei Y, Xu J . Effects of Co-Application of Diammonium Phosphate Fertilizer with Microbial Inoculant on Soil Nitrogen Levels and Alfalfa Growth Performance in Saline-Alkali Soil . Agronomy . 2026, 16 (3) .

[48] Wang W, Zhou L, He Y, Zhang Z, Zhu C, Huang Y . Synergistic promoting mechanism of modified biochar combined with functional microbial agent on saline-alkali soil improvement . Environmental Technology & Innovation . 2025, 40 .

[49] Wang X, Xia X, Riaz M, Wang J, Jiang C . Continuous biochar application ameliorates saline-alkali soils: Effects on soil multifunctionality, salt redistribution, microbial dynamics, and cotton productivity over two years . Applied Soil Ecology . 2026, 218 .

[50] Wu Q, Chen Y, Dou X, Liao D, Li K, An C, Li G, Dong Z . Microbial fertilizers improve soil quality and crop yield in coastal saline soils by regulating soil bacterial and fungal community structure . Science of The Total Environment . 2024, 949 .
[51] Xing J, Li X, Li Z, Wang X, Hou N, Li D . Remediation of soda-saline-alkali soil through soil amendments: Microbially mediated carbon and nitrogen cycles and remediation mechanisms . Science of The Total Environment . 2024, 924 .
[52] Xu D, Yu X, Zhang Y, Liu Y, Chen C, Li L, Fan S, Lu X, Zhang X . Effect of compost as a soil amendment on the structure and function of fungal diversity in saline–alkali soil . Current Research in Microbial Sciences . 2025, 9 .
[53] Yang H, Kang L, Liu Q, Li Q, Ai F, Zhang K, Zhao X, Ding K . Synergistic Effects of Coal Gasification Slag-Based Soil Conditioner and Vermicompost on Soil–Microbe–Plant Systems Under Saline–Alkali Stress . Sustainability . 2026, 18 (3) .
[54] Yang Y-M, Chen Y-K, Gong X-F, Yang J-J, Du J-H, Zhu Y . Rhizosphere regulation by three Bacillus species and tomato productivity: A feasible approach for moderately saline-alkali soil remediation . Plant Physiology and Biochemistry . 2026, 231 .
[55] Yu R, Zhang X, Zhou J, Wang W, Song J, Chang F, Wang J, Li H, Li X, Li H, Zhang H . Mitigating soil salinity–alkalinity and reshaping bacterial community to improve soil organic carbon sequestration in the Hetao Irrigation District: a combined approach of organic ameliorant and microbial agents . Frontiers in Plant Science . 2026, 17 .
[56] Zhang T, Wang X-l, Zhou J, Zhou W, Zhou S-Q . Construction of Phosphate-Solubilizing Microbial Consortium and Its Effect on the Remediation of Saline-Alkali Soil .Microbial Ecology . 2025, 88 (1) .
[57] Zhang Z, Li T, Hu R, Fu Z, Zhang Y, Ma J, Wang P, Sun J . Nitrogen-fixing bacteria and arbuscular mycorrhizal fungi alleviated nitrogen limitation in halophytes and reduced soil salinity in salt-aggregating and salt-secreting plants .Plant and Soil .2026, 520 (2): 1409-1427 .
[58] Zhao M, Zhang X, Zhang J, Zhang M, Chen X, Yang F, Dai L, Chen Y, Wang R .Combining waste biomass with functional microorganisms can effectively ameliorate hardened saline-alkali soil and promote plant growth .Plant and Soil .2025, 513 (1): 1557-1577 .
[59] Zhao W, Li S, Yang W, Cui N, Lu X, Mo S, Guo Q, Ma P . Effect of PGPRs on the Rhizosphere Microbial Community Structure and Yield of Silage Maize in Saline–Alkaline Fields . International Journal of Molecular Sciences . 2025, 26 (16) .
[60] Zhao W, Xiao J, Wang S, Gai X, Chen G . Bone biochar and humic acid improved soil quality and promoted Olea europaea growth in coastal saline soil by enhancing the stoichiometric homeostasis of nutrient elements . Biochar . 2025, 7 (1) .
[61] Zhou L, Du Z, Lv P, Wang Z, Cai C, Li J . High-concentration peat drives divergent transcriptomic responses to enhance saline-alkaline tolerance and phytoremediation in two Suaeda species . Frontiers in Plant Science . 2026, 17 .
